# Structure and Properties of Phosphate-Based Geopolymer Synthesized with the Spent Fluid Catalytic-Cracking (SFCC) Catalyst

**DOI:** 10.3390/gels8020130

**Published:** 2022-02-18

**Authors:** Qian Wan, Ruobing Zhang, Yimin Zhang

**Affiliations:** 1 School of Resource and Environmental Engineering, Wuhan University of Science and Technology, Wuhan 430081, China; zhangrb1129@163.com; 2 State Environment Protection Key Laboratory of Mineral Metallurgical Resources Utilization and Pollution Control, Wuhan University of Science and Technology, Wuhan 430081, China; 3 Collaborative Innovation Center of Strategic Vanadium Resources Utilization, Wuhan 430081, China; 4 Hubei Provincial Engineering Technology Research Center of High Efficient Cleaning Utilization for Shale Vanadium Resource, Wuhan 430081, China

**Keywords:** phosphate-based geopolymer, SFCC catalyst, mechanical property, microstructure

## Abstract

As a common industrial by-product, the spend fluid catalytic-cracking (SFCC) catalyst was used to prepare phosphate-based geopolymer for the first time. The structure and property of geopolymer with phosphoric acid concentration ranging from 6 to 14 mol/L was characterized by compressive strength measurements, X-ray powder diffraction (XRD), Fourier Transform Infrared spectroscopy (FTIR), scanning electron microscopy (SEM), and ^27^Al and ^29^Si nuclear magnetic resonance (NMR). A stable binder was formed with the compressive strength in the range of 9.8 to 30.2 MPa when the acid concentration was between 6 and 12 mol/L. The higher concentration of acid can promote the dissolution of raw materials and formation of geopolymer gels. The coordination of silicon and aluminum in geopolymer gel synthesized with the SFCC catalyst and metakaolin is similar. Compared with the geopolymer with metakaolin, which forms more Si-O-Al bonds, in the networks of geopolymer with the SFCC catalyst, more Si(Al)-O-P bonds were formed. These results indicate that the SFCC catalyst can be an excellent raw material for the synthesis of phosphate-based geopolymer.

## 1. Introduction

Geopolymer is considered to be a green building material that can replace Portland cement and attract increasing attention [1]. It is synthesized through activating the aluminosilicate source such as metakaolin with an activator solution [2]. According to the type of activator, it can be divided into two categories, namely alkali-based and acid-based geopolymers, resulting, respectively, from activation in alkaline and acid (phosphoric acid and humic acid) environments [3,4]. Alkali-based geopolymers are composed of cross-linked tetrahedral [AlO_4_]^4−^ and [SiO_4_]^4−^ units [5], but in the microstructure of acid-based geopolymers, [SiO_4_] ^4−^ is partially replaced by [PO_4_]^5−^ to form a Si-Al-P binder system, which has stronger bonding and exhibits better properties, including higher compressive strength, lower efflorescence, and higher thermal stability [6,7,8]. Therefore, phosphate-based geopolymer can be used in cement-based construction as well as in the adsorption and immobilization of toxic metals [9,10]. The raw materials for the phosphate-based geopolymer mainly come from some calcined clay (e.g., metakaolin [11], laterite [12], and halloysite [6]). As natural minerals, the supply of these precursors is limited, which would be an obstacle in large-scale production and application. It is necessary to identify different precursors with similar chemical properties in order to extend the raw materials of phosphate-based geopolymer from natural minerals to other materials, such as industrial waste.

The spent fluid catalytic-cracking (SFCC) catalyst is a kind of aluminosilicate material which obtains, as a byproduct from petroleum, fluidized cracking production [13]. The annual production of the SFCC catalyst in the world is about 160,000 tons and the main disposal method is land fill deposition, which not only leads to high costs ($200/ton) but also to a waste of resources [14,15]. The previous studies have used catalysts as alternative materials to replace a part of cement to produce concrete and achieved good results [16,17]. However, in recent years, considering the pressure of environmental protection [18,19] and the requirement of carbon emission [20], some studies have turned to investigating the feasibility of using SFCC catalysts to prepare geopolymers, the energy consumption and CO_2_ emissions of which are 70% and 80% lower than that of cement, respectively [21,22]. SFCC catalysts mainly consists of silicon and aluminum, which has good potential as a raw material for the synthesis of geopolymer. Zhang et al. [23] used the SFCC catalyst as a replacement of metakaolin to synthesize geopolymer and the highest compressive strength was 41.22 MPa. Bouzón et al. [24] synthesized geopolymers with the SFCC catalyst and rice husk ash. The result showed that the compressive strength of geopolymer was in the range of 31–41 MPa. Zhang et al. [25] only used the SFCC catalyst as a raw material to synthesize geopolymer and the influences of the particle size of the SFCC catalyst, type of activator agent solutions, and mass ratios of SiO_2_/Na_2_O on the compressive strength were investigated. For the final product, the highest strength was not higher than 4 MPa. These studies have shown that the SFCC catalyst can completely or partially replace raw materials to prepare alkali-activated geopolymers; however, when it is completely used as a raw material, the compressive strength of the product is low and some highly active raw materials (metakaolin or rice husk ash) need to be added as additives to prepare geopolymers with satisfactory strength. In addition, there are no reports about its preparation of phosphate-based geopolymers.

Therefore, in this work, we investigated the feasibility of the preparation of phosphate-based geopolymer by using the SFCC catalyst. The microstructure of geopolymer prepared with different concentrations of phosphoric acid was investigated and compared with the microstructure of conventional phosphate-based geopolymer synthesized with metakaolin. This study not only expands the possibilities for raw materials for the synthesis of phosphate-based geopolymer but also gives a further understanding about the effect of raw materials on the structure of geopolymer.

## 2. Experimental Setup

### 2.1. Materials

**Figure 1:**
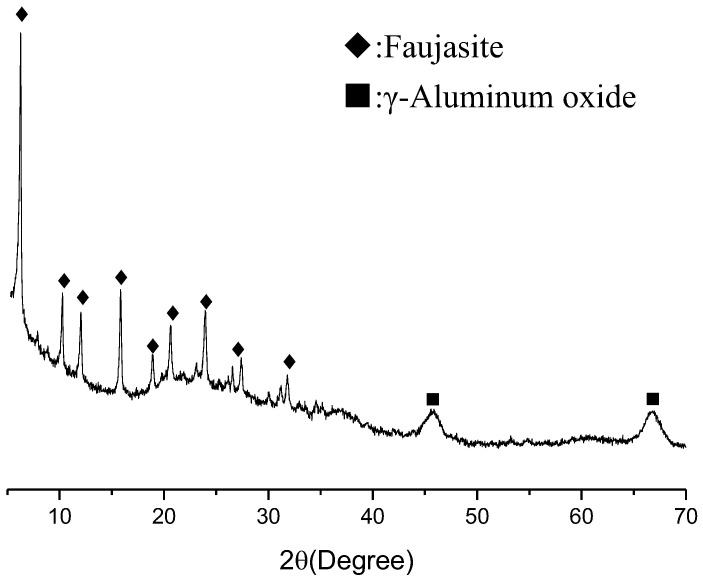
XRD pattern of spent fluid catalytic-cracking catalyst.

The SFCC catalyst obtained from SINOPEC-SK(Wuhan) Petrochemical Company Limited was used as a raw material. A pretreatment of ball mill was preformed for 15 min to increase its reactivity. It measured the particle size range of 0.5–37.54 μm with a mean particle diameter of 8.8 μm by a Shimadzu SALD-1100 laser diffraction analyzer. Figure 1 shows the X-ray diffraction pattern of the SFCC catalyst, wherein the main phase is faujasite (reference code 96-154-0288) with a small amount of γ-Aluminium oxide (reference code 96-101-0462). Table 1 gives the chemical analysis of the SFCC catalyst measured by XRF (X-ray fluorescence spectroscopy), in which the main components are Al_2_O_3_ and SiO_2_. The phosphoric acid (H_3_PO_4_, industrial grade) with 85% purity was used the activator in the synthesis. Deionized water was used in all experiments.

### 2.2. Synthesis of Geopolymer

The phosphate-based geopolymer was synthesized through activating raw materials by phosphoric acid solution with different concentrations. At first, the phosphoric acid was mixed with deionized water to prepare the phosphoric acid solution with molar concentrations at 6, 8, 10, 12, and 14 mol/L. Solutions were stored for 24 h prior to use. The raw materials were activated by obtained solution and stirred for 5 min to the homogeneous mixture. Then, the blend was poured into a 5 cm × 5 cm × 5 cm polyethylene mold and the mold was vibrated on a vibration table to release the air bubble. After being sealed with polyethylene film, it was first cured at 60 °C for 6 h and continued at room temperature for 7 days. A product synthesized with metakaolin as a raw material and 12 mol/L as the activator were used for comparison. A diagram of the geopolymer preparation process is shown in Table 2. 

### 2.3. Characterization

The compressive strength of geopolymers was measured by a mechanical tester (Hangzhou Xingo Technology EHC-1300) based on ASTM standards ASTM C109 [26]. In the measurements of compressive strength, at least three specimens were tested and the average value was used. The mineralogical study and structure of geopolymer was characterized by X-ray diffraction (XRD, Brucker D8) with Cu Ka radiation, scanning electron microscopy (SEM, JEOL JSM-5610LV), and Fourier transform infrared spectroscopy (FTIR, NexusJSM-5610). ^29^Si and ^27^Al nuclear magnetic resonance (NMR, Bruker AVANCE III) spectra were used to analyze the microstructure of geopolymer gel. ^29^Si NMR spectra were collected at 79.5 kHz on a 7 mm probe with a spinning speed of 5 kHz, a pulse width of 6.5 μs, and a relaxation delay of 10 s. ^27^Al NMR spectra were obtained using a 4 mm probe at 156 MHz with a pulse width of 6.5 μs, spinning speed of 41.7 kHz, and relaxation delay of 2 s. The ^29^Si and ^27^Al chemical shifts were referenced to an external standard of tetramethylsilane and Al(NO_3_)_3_ solution.

## 3. Results and Discussion

**Figure 2:**
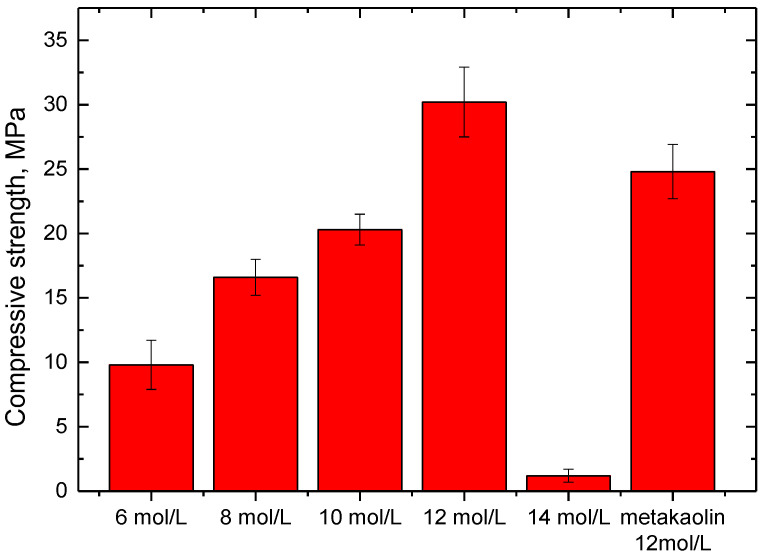
Compressive strength of geopolymer synthesized with different conditions.

Figure 2 presents the compressive strength of phosphate-based geopolymer synthesized with different conditions. When the phosphoric acid concentration was 12 mol/L, the SFCC catalyst-based geopolymer obtained the highest compressive strength of 30.2 MPa, which is even higher than that of metakaolin-based geopolymer with the same condition. This suggests that the SFCC catalyst can be an excellent raw material for the preparation of phosphate-based geopolymer. Compared with the preparation of alkali activation, activating the SFCC catalyst with phosphoric acid can achieve satisfactory strength without adding high-activity additives. With the acid concentration decreasing from 12 to 6 mol/L, the compressive strength of geopolymers decreased from 30.2 MPa to 9.8 MPa. However, at a higher acid concentration of 14 mol, it almost could not condense and harden to form geopolymer, and the compressive strength was only 1.2 MPa. This result demonstrates that the concentration of phosphoric acid has a significant effect on the mechanical properties of geopolymer synthesized with the SFCC catalyst. Increasing the concentration of phosphoric acid can increase the dissolution of silica and alumina from the SFCC catalyst and promote the formation of geopolymer gel, but excessive phosphoric concentration with high viscosity will hinder the depolymerization of raw materials and negatively affect the compressive strength of the product.

**Figure 3:**
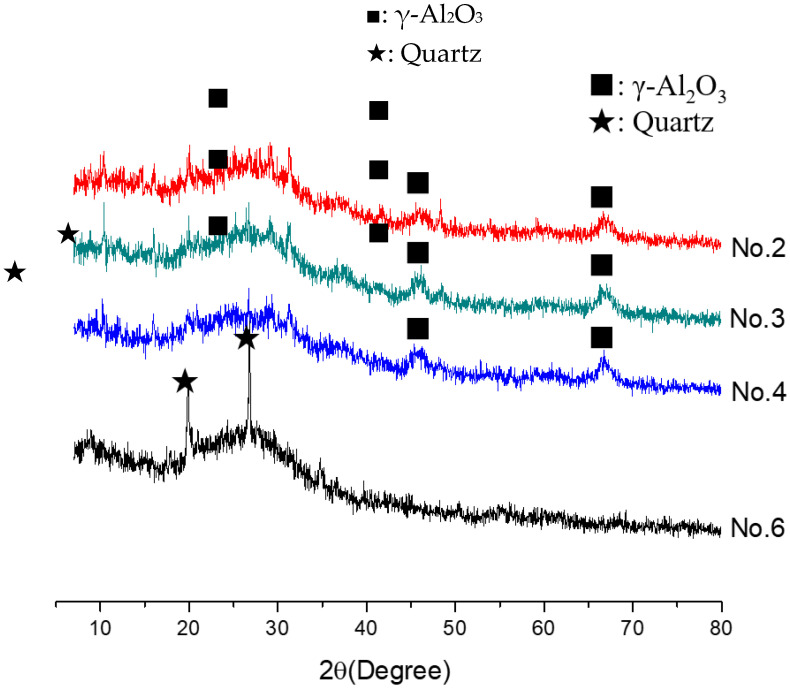
XRD pattern of geopolymer synthesized with different conditions.

**Figure 4:**
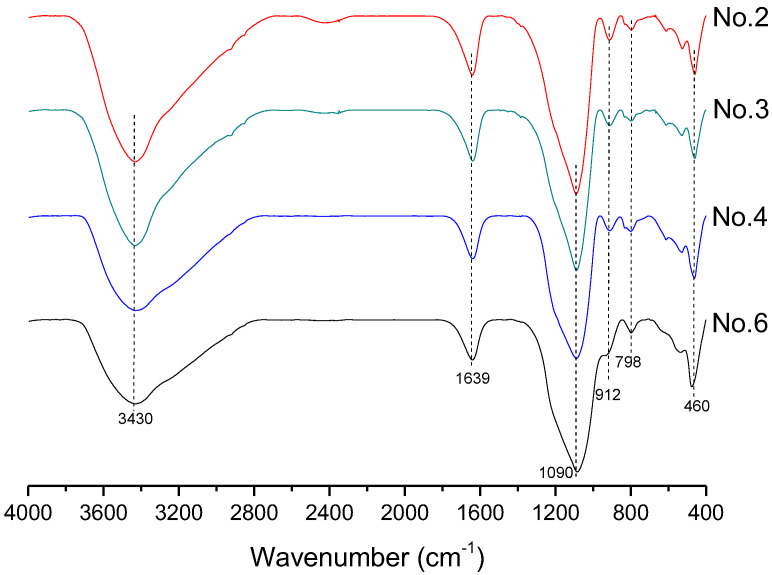
FTIR spectra of geopolymer synthesized with different conditions.

In order to investigate the microstructure of different geopolymers, the XRD and FTIR analysis was performed. Figure 3 presents the XRD pattern of geopolymer synthesized with different conditions. In the geopolymer synthesized with the SFCC catalyst, the peak of Faujasite in raw materials disappeared and the diffuse hump between 18 and 30° centered around 22° shifted to 18 and 34° as well as centered at 28°, which is a typical diffuse halo structure also existing in phosphate-based geopolymer synthesized with metakaolin [3]. This indicated that the SFCC catalyst can be activated by phosphoric acid and form geopolymer. The intensity of the hump increased with the increase of the concentration of phosphoric acid, suggesting that the higher concentration of phosphoric acid promotes the depolymerization of raw materials as well as the formation of geopolymer gel. The diffraction peaks of quartz and aluminum oxide are derived from raw materials, which do not participate in the geopolymerization. The FTIR spectra of SFCC-based geopolymer synthesized with different phosphoric acid concentrations are shown in Figure 4. The broad band appeared at 1639 cm^−1^ and 3430 cm^−1^ is the H-O-H and O-H stretching vibrations of free water [27]. The peaks at 460 cm^−1^, 798 cm^−1^, 912 cm^−1^, and 1090 cm^−1^ correspond to the bending vibration of Si-O [28], stretch vibration of Si-O-Si [29], and the stretching of Si-O-P [30], and overlap between the bands of the Si-O vibration, P-O vibration, and Al-O vibration [31]. The intensity of all characteristic peaks in the XRD and FTIR of different geopolymers is similar, indicating that the SFCC catalyst can be used as an alternative raw material for metakaolin to produce phosphate-based geopolymers and the geopolymer synthesized with the SFCC catalyst has a similar structure to that with metakaolin. 

**Figure 5:**
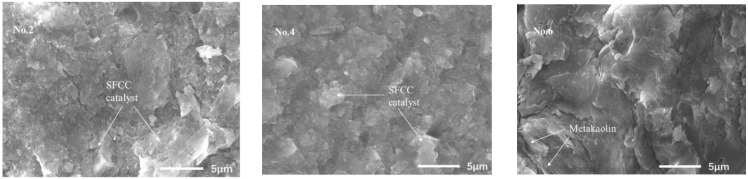
SEM images of geopolymers synthesized with different acid concentrations.

Figure 5 presents the SEM images of geopolymers synthesized with different acid concentrations, which were analyzed at 5000× magnification. For the sample of No. 4, the structure of the SFCC catalyst is damaged more seriously and a more homogeneous geopolymer binder is formed than in No. 2. This indicates that a higher concentration of acid can promote the dissolution of raw materials and the formation of geopolymer gels, thereby increasing the compressive strength of geopolymer. For the samples of No. 6, a homogenous structure also was formed but the layered metakaolin remained in the gel, causing a lot of crack formation. This may be the reason for the higher strength of SFCC catalyst-based geopolymer than for metakaolin-based geopolymer.

**Figure 6:**
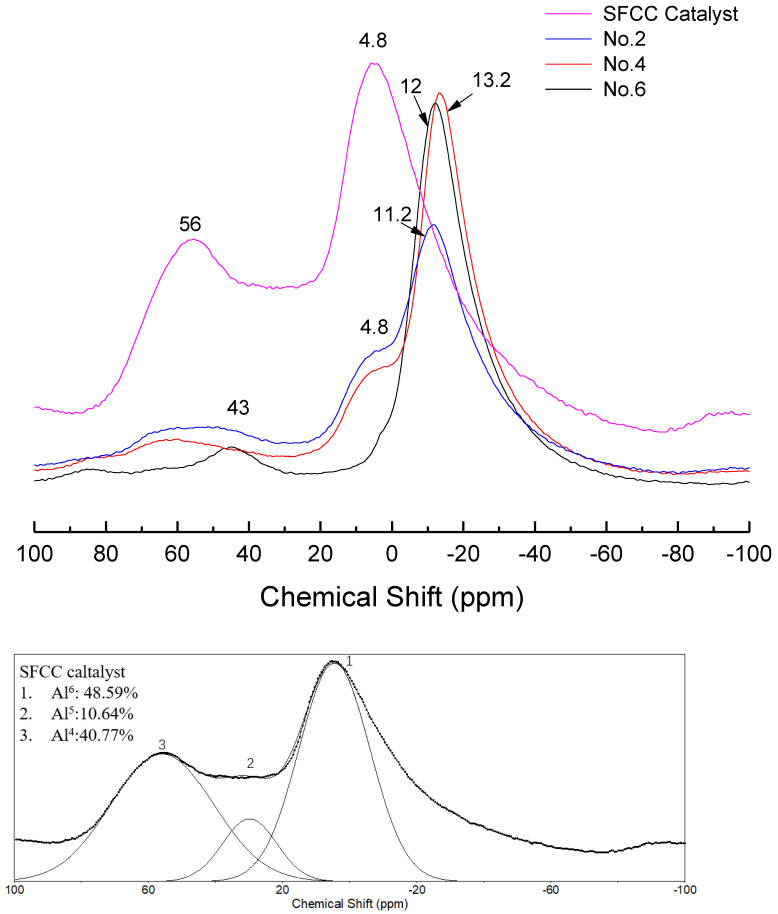

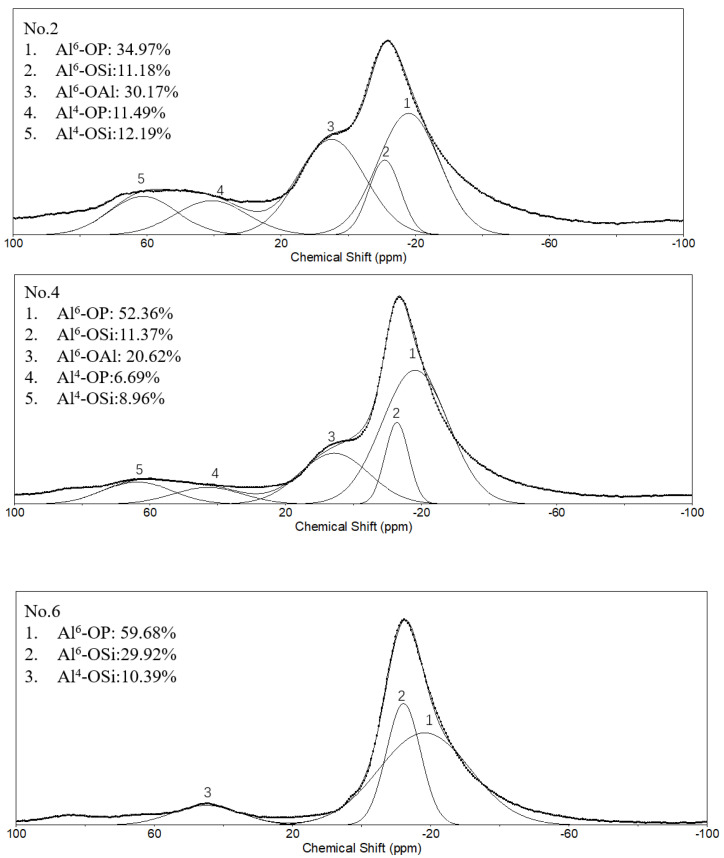
^27^Al NMR spectra and their deconvolution of SFCC catalyst and phosphate-based geopolymer.

In order to further study the differences in the molecular structure of geopolymers with different conditions, the NMR analysis was performed. Figure 6 shows the ^27^Al NMR spectra of different geopolymers and SFCC catalysts. The spectra of the SFCC catalyst exhibited two sharp peaks at 4 ppm and 58 ppm, which are attributed to Al^VI^ and Al^IV^ in the Si-O-Al-ordered structure [32]. This deconvolution reveals the presence of another resonance at 27 ppm, which corresponds to the Al^V^ structure. A new peak formed at the chemical shift between 0 and −20 ppm, which is the characteristic peak of phosphate-based geopolymer, corresponding to Al^VI^ in Al(OP)_6−x_(SiO)_x_ [33]. According to previous studies, this deconvolution reveals the presence of two resonances [34]. The resonance located at −12 ppm is attributed to Al^VI^ in condensed aluminosilicate networks and the other one located at −18 ppm is assigned to Al^VI^ in polymerized phosphate aluminum networks. When the phosphoric acid activator was added, the characteristic peak for the SFCC catalyst was obviously weakened and a shoulder peak remained at 4 ppm, corresponding to six-coordinated aluminum species of γ-Al_2_O_3_ [35], which came from the raw material. This is also consistent with the results of XRD, in which only part of γ-Al_2_O_3_ can be dissolved and participate in the geopolymer gel, and most of it still remained in the geopolymer. As the acid concentration increased from 8 to 12 mol/L, the content of Q^4^ in SFCC and Q^6^ in γ-Al_2_O_3_ decreased and the content of Al^6^-OP increased. The results show that most SFCC catalysts can depolymerize in the activator and with the increase of the acid concentration, more P is involved in the aluminum hexahedron of geopolymer and more Al-O-P bonds are formed. In addition, for geopolymer synthesized with the SFCC catalyst, the ratio of Al^6^-OP to Al^6^-OSi was close to 3:1 in No. 2 and 5:1 in No.4, but for geopolymer prepared with metakaolin, it was close to 2:1. This indicates that more Al-O-P bonds are formed in geopolymer prepared by the SFCC catalyst, while in geopolymer with metakaolin, more Si-O-Al bonds are formed.

**Figure 7:**
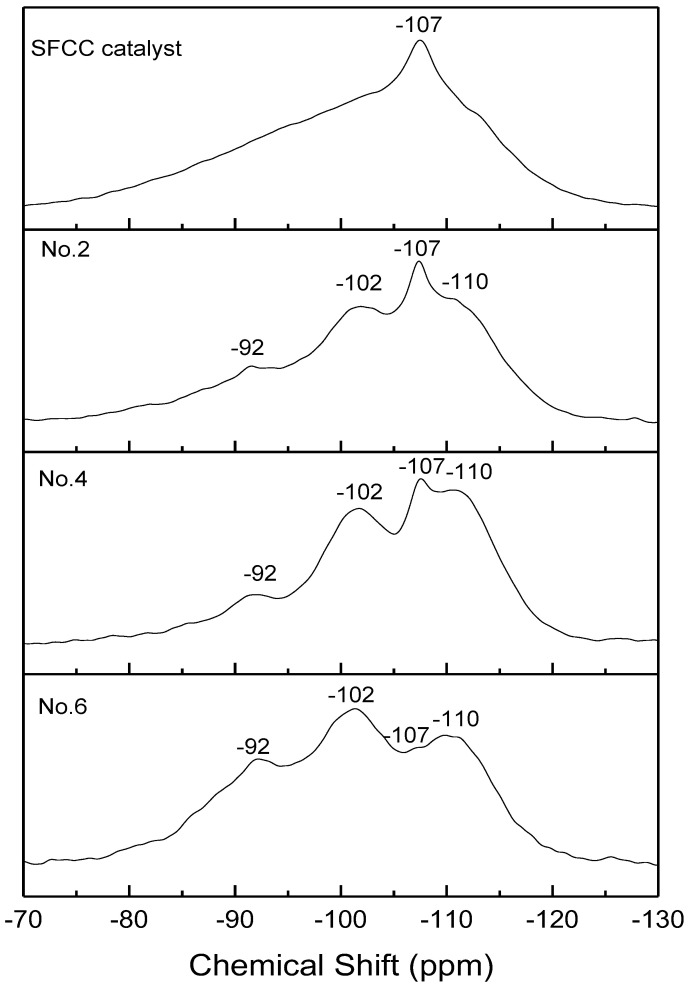
^27^Si NMR spectra of SFCC catalyst and phosphate-based geopolymers.

The ^29^Si NMR spectra of the SFCC catalyst and different geopolymer are shown in Figure 7. All the spectra showed three broad signals centered at −110, −102, and −92 ppm, which are related to Q^4^(3Si, 1P), Q^4^(1Al) [30], and Q^4^(2Al) [31]. This also confirms that the microstructure of geopolymer prepared by the SFCC catalyst is similar to that prepared by metakaolin. Compared with geopolymer prepared by metakaolin, in SFCC catalyst-based geopolymer, the intensity of Q^4^(3Si, 1P) is stronger and the intensity Q^4^(2Al) is low, indicating that SFCC geopolymer has more Si-O-P bonds and relatively less Si-O-Al bonds. This is also consistent with the results of ^29^Al NMR that more Si-O-Al bonds are formed in geopolymer prepared by metakaolin. With the acid concentration increasing from 8 mol/L to 12 mol/L, the intensity of the Q^4^(2Al) peak decreased and the intensity of Q^4^(3Si, 1P) and Q^4^(1Al) increased. This indicates that the increase in the acid concentration promotes the decomposition of the SFCC catalyst, resulting in more Si and P participating in the geopolymer structure. The narrow peak at −107 ppm was observed in the spectra of the SFCC catalyst due to Q^4^(0Al) sites of USY zeolites [36]. It is a common characteristic peak in the spectra of the SFCC catalyst, which also exists in the synthesized geopolymer, indicating that part of the USY zeolites has difficulties participating in the geopolymerization and remains in geopolymer.

## 4. Conclusions

SFCC catalyst as a kind of industrial solid waste can be used as an excellent raw material for the preparation of phosphate-based geopolymer. The concentration of phosphoric acid has a significant effect on the mechanical properties of geopolymer synthesized with the SFCC catalyst. When the acid concentration is between 6 and 12 mol/L, a stable binder was formed with a compressive strength in the range of 9.8 to 30.2 MPa. The higher concentration of acid can promote the dissolution of raw materials and formation of geopolymer gels, thereby increasing the compressive strength of geopolymer. The SFCC catalyst can be used to synthesize phosphate-based geopolymer with a similar structure to that of metakaolin. However, in the geopolymer network, for geopolymer synthesized with the SFCC catalyst, with a concentration of acid increase, the ratio of Al^6^-OP to Al^6^-OSi is 3:1 to 5:1, but for geopolymer prepared with metakaolin, it is close to 2:1. The former has more Al-O-P bonds and Si-O-P bonds, while the latter has more Si-O-Al bonds.

## Figures and Tables

**Table 1:** Chemical composition of the SFCC catalyst.

Component	SiO_2_	Al_2_O_3_	Fe_2_O_3_	CaO	K_2_O	Na_2_O
wt%	37.63	55.29	0.58	0.39	0.21	0.15

**Table 2:** Preparation regime of phosphate-based geopolymers.

Specimen No.	Raw Materials		Acid Volume	Acid Concentration (mol/L)
	SFCC Catalyst (g)	Metakaolin		
1	50 g	0 g	40 mL	6 mol/L
2	50 g	0 g		8 mol/L
3	50 g	0 g		10 mol/L
4	50 g	0 g		12 mol/L
5	50 g	0 g		14 mol/L
6	0 g	50 g	40 mL	12 mol/L

## Data Availability

The data presented in this study are available from the corresponding author upon request.
